# Parental Opinion about the Low FODMAP Diet in Dietary Treatment of Children with Functional Abdominal Pain

**DOI:** 10.3390/ijerph17155554

**Published:** 2020-07-31

**Authors:** Katarzyna Mirosława Boradyn, Elżbieta Jarocka-Cyrta, Katarzyna Eufemia Przybyłowicz, Małgorzata Obara-Gołębiowska

**Affiliations:** 1 Department of Human Nutrition, Faculty of Food Sciences, University of Warmia and Mazury in Olsztyn, Słoneczna 45F, 10-718 Olsztyn, Poland; katarzyna.przybylowicz@uwm.edu.pl; 2 Department of Pediatrics, Gastroenterology and Nutrition, Faculty of Medicine Collegium Medicum, University of Warmia and Mazury in Olsztyn, Żołnierska 18A, 10-561 Olsztyn, Poland; elzbieta.jarocka@uwm.edu.pl; 3 Department of Clinical, Developmental and Educational Psychology, Faculty of Social Sciences, University of Warmia and Mazury in Olsztyn, Prawocheńskiego 13, 10-447 Olsztyn, Poland; m.obara-golebiowska@uwm.edu.pl

**Keywords:** functional abdominal pain, pediatric, diet therapy, FODMAP, children, parents

## Abstract

The aim of this study was primarily to evaluate differences between parental opinion about the diet and overall changes in children’s symptoms of functional abdominal pain (FAP) during the low fermentable oligosaccharides, disaccharides, monosaccharides, and polyols (FODMAP) diet and National Institute for Health and Care Excellence (NICE) diet. Secondly, this paper examined the agreement between parental perception of children’s symptoms and children’s self-assessment of symptoms during the diet in both treatment groups. Twenty-seven children with diagnosed functional abdominal pain (FAP) were randomized to one of two group, receiving the low FODMAP diet or the diet based on NICE guidelines. Children reported gastrointestinal symptoms at baseline and during the diet. At the end of the intervention, parents assessed their children’s diet and symptoms changes, using Likert scales. The agreement between parental and children assessments of gastrointestinal symptoms was defined as the percentage of compatible answers. In the low FODMAP group a significantly lower percentage of parents (38%) declared that it was easy to follow the diet, compared to the NICE group (57%), (*p* = 0.017). A high percentage of parents in both groups reported improvement in all symptoms of children during dietary intervention. A high level of agreement was also observed between parental and children’s self-assessment of abdominal pain intensity and frequency. Our research suggests that in parental opinion the low FODMAP diet is as effective as the diet based on NICE guidelines in children with FAP. However, the low FODMAP diet may seem more difficult to follow, and this may have had an impact on the effectiveness and acceptability of the FODMAP diet by children.

## 1. Introduction

Functional abdominal pain disorders (FAPDs) such as functional abdominal pain (FAP), irritable bowel syndrome (IBS), functional dyspepsia (FD), and abdominal migraine (AM) are common health problems in childhood and adolescence [1,2]. Etiology and pathophysiology of FAPDs are still unclear and there is no effective treatment method [3]. 

Current studies have indicated that certain foods can be contributing factors exacerbating symptoms of FAPDs and emphasized that dietary intervention is an important non-pharmacological treatment option [4]. An increased interest has been observed in a new diet which recommends reducing the intake of fermentable oligosaccharides, disaccharides, monosaccharides, and polyols (FODMAP). These short-chain carbohydrates are poorly absorbed, osmotically active, and rapidly fermented by bacteria, which can trigger symptoms of FAPDs [5]. The second frequently used dietary approach is based on lifestyle guidance developed by National Institute for Health and Care Excellence (NICE) for IBS patients (as a type FADPs) [6]. NICE dietary recommendations focus mainly on regular meal patterns, appropriate portion sizes, mealtimes atmosphere, adequate fiber and fluid intake, avoidance of sweets and fizzy beverages [3,4]. 

Nevertheless, patients with FAPDs often try to identify certain foods and triggering factors themselves and attempt to introduce new diets found on the internet or eliminate certain products from the diet [5,7]. This may increase the risk of nutritional deficiencies and developing unhealthy eating habits, which especially in children are undesirable practices [5]. Moreover, the effectiveness of diets used without consulting a dietitian may be unsatisfactory and difficult to follow due to insufficient understanding of the recommendations [4,8,9].

Without effective treatment recurrent symptoms of the FAP may have a significant impact not only on children’s quality of life, but also parents and the entire family [1,2,3]. Numerous medical visits and intensification of a child’s gastrointestinal symptoms may generate stress and absences of parents at work. In addition, research suggests that the emotional bond between parents and children can often affect the parental assessment of symptoms and thus increase anxiety in both parties [1,10]. The parental perception of the disease may also influence a child’s illness experience [10,11,12]. Moreover, excessive parental focus on a child’s health condition as well as increased tension and anxiety of parents may contribute to the higher frequency of reported symptoms in children [10,11,13]. 

The aim of this randomized controlled trial was primarily to evaluate differences between parental opinion about the diet and overall changes in children’s symptoms of functional abdominal pain in two treatment groups: The low FODMAP and NICE diet. Secondly, this paper examined the agreement between parental perception of children’ symptoms and children’s self-assessment of symptoms during the diet in both treatment groups.

## 2. Materials and Methods 

### 2.1. Participants 

Eligible patients referring to the Department of Pediatrics, Gastroenterology, and Nutrition, aged 5–12 years who met Rome III criteria for FAP [14] participated in this study between January and October 2017. Parents or legal guardians of children were informed about the objectives of the study and signed an informed consent form. Exclusion criteria were: Organic gastrointestinal tract disorders; presence of another type of FAPDs (IBS, FD, or AM); known food allergies; chronic inflammatory gastrointestinal disorders; acute infection; and any antibiotic treatment within the last 8 weeks.

### 2.2. Study Protocol

This was a double-blind, randomized, controlled trial conducted in line with CONSORT guidelines. Participants were randomized according to a random number table and stratified by age to receive 28 days of the low FODMAP diet or the diet based on NICE guidelines as a control diet. Children and study dietitian were blinded to the diets. All food was prepared and provided by a catering company. Patients reported gastrointestinal symptoms during the 2-week baseline period and during the four weeks of dietary intervention. Parents were informed that they could contact the study dietitian at any time during the dietary intervention in case of any concerns. On the fourteenth day of the intervention, the dietitian contacted parents to monitor and assess any potential problems or address any dietary or health concerns. On the last day of the intervention during the summary meeting, parents completed questionnaires about their opinions on the diet and changes in children’s symptoms during the intervention. The timeline of the study is shown in Figure S1.

The study was approved by the Bioethics Committee of the Faculty of Medical Sciences at the University of Warmia and Mazury in Olsztyn (Resolution No. 6/2015) and was registered on ClinicalTrials.gov (identifier NCT03771027). 

### 2.3. Interventional Diets

Both diets were developed by a study dietitian in accordance with the Human Nutrition Recommendations for Polish Population [15]. A daily diet consisting of three main meals and two snacks was delivered by a catering company to the participants each morning. However, parents were instructed that children eat accordingly to their appetite and to record any additional products or meals that were consumed. To facilitate compliance with the diet, three food lists were provided with: (i) Products that are allowed, (ii) products that should be avoided, (iii) additional products allowed only in limited amounts. The low FODMAP diet was based on the Monash University recommendations contained in the Low FODMAP Diet AppTM (Melbourne, Victoria, Australia) [16]. Included products were divided into three groups and labeled accordingly to the level of FODMAP content: (1) Low—recommended in larger amounts (2) moderate—recommended in small amounts, and (3) high—only limited amounts or none to include in the diet. The control diet based on NICE guidelines was composed of products with different FODMAP contents. An example of the daily menu for each group is shown in Table S1. 

### 2.4. Questionnaires

1. The frequency of abdominal pain was assessed with Wong–Baker FACES pain rating scale. The tool uses a series of facial expressions to illustrate a spectrum of abdominal pain intensity. The overall score of the scale ranges from 0 = no pain to 10 = hurts the worst [17]. The frequencies of abdominal pain were recorded as number of events reported per day by children.

2. The Bristol stool form scale was used to assessed stool consistency. The assessment was made by children. This diagnostic medical tool classifies the form of human feces into seven categories. These types were combined into three groups: Hard or lumpy stool—type 1 or 2; normal stool—type 3–5 and type 6 or 7: Loose or watery stool—type 6 or 7 [18].

3. Abdominal pain intensity and frequency as well as stool consistency were assessed by children using the questionnaires described above during baseline and dietary intervention. Changes of gastrointestinal symptoms were rated as improvement or no improvement.

4. A 7-point Likert scale was used to examine parents’ opinions about impact of dietary intervention on symptoms in children. The answers were divided into two groups of estimated changes in symptoms either as an improvement or no improvement [4]. 

5. A 5-point Likert scale was used to examine parents’ opinions on: (i) Understandability of diet recommendations obtained from dietitian before dietary intervention, (ii) ease of following a diet in the context of: Product availability, acceptability by children, and difference from usual diet, and (iii) satisfaction with the diet effects. The answers were scored from 1 = strongly disagree to 5 = strongly agree. Responses were collapsed into agree, neither agree nor disagree, and disagree responses [4,19]. Both questionnaires with the Likert scale were completed after the dietary intervention by parents. 

### 2.5. Assessment of the Agreement on Symptoms Perception

Children’s self-assessment of symptoms during the diet was compared to parental perception of children’s symptoms changes in the low FODMAP group and in the NICE group. The results were presented as a percentage of compatible assessments regarding the intensity and frequency of abdominal pain and stool consistency in each group.

### 2.6. End Points

The primary end point was the differences in parental opinion about the diet and overall symptom changes in their children on the low FODMAP diet compared with the NICE diet.

A secondary end point included the evaluation of the agreement between parental perception of children’ symptoms and children’s self-assessment of symptoms during the diet in both groups.

### 2.7. Statistical Analysis

Statistical analyses were carried out using STATISTICA software (version 10.0 PL; StatSoft Inc., Tulsa, OK, USA; StatSoftPolska, Kraków, Poland). Continuous data are presented as mean ± SD, and categorical variables as sample percentages (%). The differences between groups were verified using chi-square test. Differences were considered significant for *p* < 0.05. 

## 3. Results

**Figure 1:**
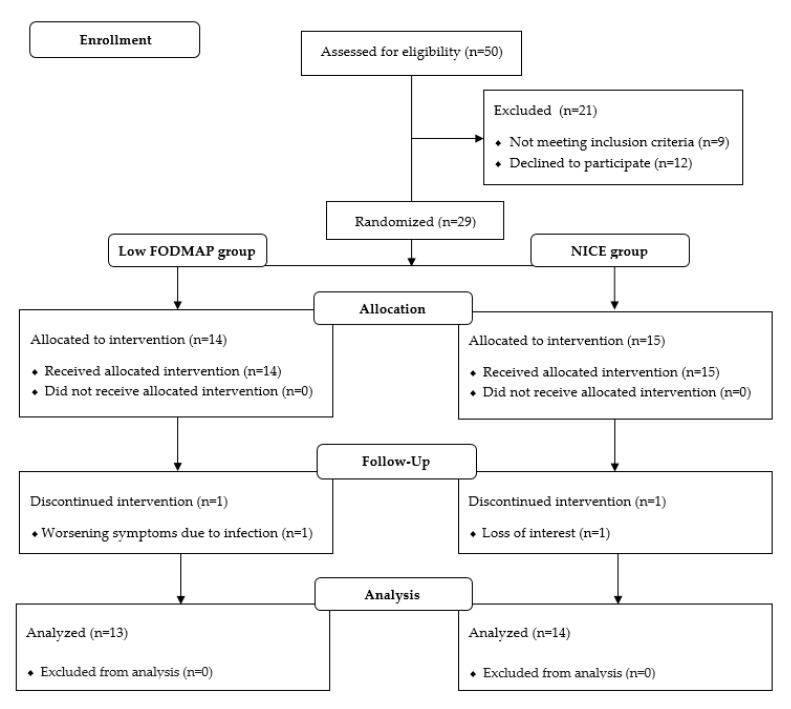
Diagram of the enrolment, randomization, and study allocation.

Fifty patients entered the screening period and were assessed for eligibility. Out of the 29 who met the eligibility criteria, 14 were randomized to the low FODMAP group and 15 to the NICE group. In the low FODMAP group, one patient had resigned because of worsening of the symptoms due to infection. In the NICE group, one patient resigned during follow-up because of loss of interest. Therefore, the final data was obtained from 13 patients in the low FODMAP group and 14 patients in the NICE group (Figure 1).

Baseline characteristics and children’s self-assessment of gastrointestinal symptoms in the two groups are summarized in Table 1. The groups were similar in terms of age, gender, and BMI z-score. There were no significant differences in children’s self-assessment of gastrointestinal symptoms between groups during the dietary intervention. Therefore, a tendency toward a lower percentage of children reporting improvement in symptoms was observed in the low FODMAP group compared to the NICE group.

**Figure 2:**
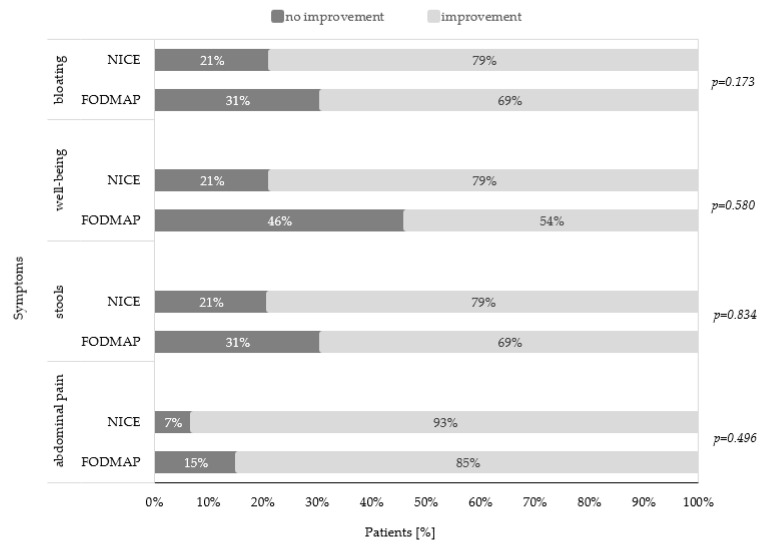
Parental assessment of children’s symptoms in the low fermentable oligosaccharides, disaccharides, monosaccharides, and polyols (FODMAP) group and National Institute for Health and Care Excellence (NICE) group after dietary intervention.

Parental assessment of the change in children’s symptoms during dietary intervention according to received diet is shown in Figure 2. A high percentage of parents reported improvement in all symptoms of children during dietary intervention in both groups. However, no significant differences were detected between the groups.

A high agreement between parental assessment and children’s self-assessment of abdominal pain intensity and frequency during the diet was observed in both groups. In the low FODMAP diet, a tendency for a lower agreement in the assessment of stool consistency was observed compared to the NICE group, but without statistical significance (Table 2).

**Figure 3:**
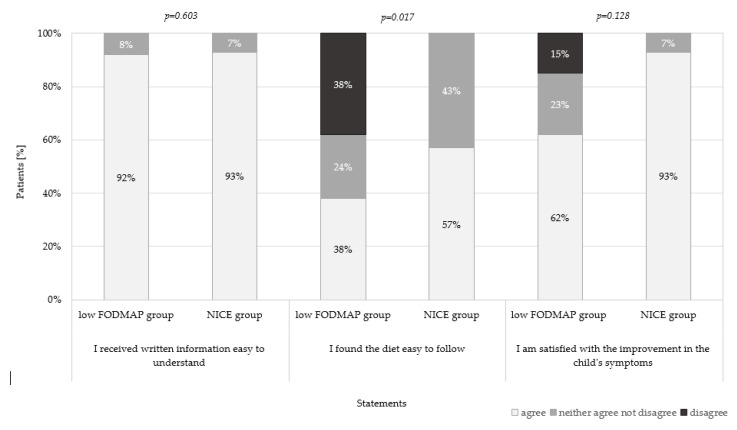
Parental agreement with opinion statements about the received diet.

In the low FODMAP group a significantly lower percentage of parents (38%) declared that it was easy to follow the diet, in comparison to the NICE group (57%), and 38% of parents additionally disagreed with this statement (*p* = 0.017). There were no significant differences between the groups when reporting the easiness of the understanding of written information (the low FODMAP group 92% versus the NICE group 93%, *p* = 0.603) and satisfaction with the improvement in a child’s symptoms (the low FODMAP group 62% versus the NICE group 93%, *p* = 0.128) (Figure 3).

## 4. Discussion

This randomized controlled trial is the first study examining parents’ opinions about the dietary intervention in children with FAP. It is also the first study evaluating the agreement between children’s and parental assessment of gastrointestinal symptom changes in children with FAP during dietary intervention. Our data demonstrated that the NICE diet was perceived as easier to follow than the low FODMAP diet. The majority of parents reported improvement in most children’s symptoms during dietary intervention in both groups. Nevertheless, we found that the greater degree of agreement between parental and children’s assessment of symptoms changes was in the low FODMAP group.

The understanding of dietary recommendations is the key to a successful dietary intervention. The low FODMAP diet is a relatively novel form of dietary treatment in FAPD, which is gaining increasing popularity and availability of information. Unfortunately, due to the evolving nature of the diet (e.g., continuous revisions of the recommendations), a lot of this information and the educational materials in magazines and internet is incomplete or simply unclear to the general audience [8,9]. Therefore, the study evaluated parents’ opinions about the diet received by their child during the intervention.

Results of our study showed that almost all parents in both groups identified the written information about the diet as easy to understand. However, in the low FODMAP group, a significantly lower percentage of parents agreed with the statement, “I found the diet easy to follow”, compared to the NICE group. In addition, more than a third of respondents in this group disagreed with this statement, considering the diet as difficult. There are only a few studies which examined the opinion and satisfaction of the diet among patients [4,20], and only one in children [21]. In the latter study, children (4–17 years) followed the low FODMAP diet for four weeks. The intervention was based on recommendations provided by a dietitian through dietary consultations and a self-catered diet. In contrary to our findings, most children defined the diet as easy to follow [21]. Perhaps the reason was the older age of the children, which may affect the lifestyle, perception of following the diet, or food choices. In addition, in a study conducted among adults with IBS, a high level of agreement was observed with statement “I found diet easy to follow” among the participants assigned to the low FODMAP diet (70%), but without statistical differences to a standard diet (85%) [4]. Another study conducted by O’Keeffe et al. (2017) examined the components of dietary acceptability among patients with IBS [20]. The results showed that most patients in the “adapted FODMAP” group defined the diet as more expensive than a standard diet. Moreover, a higher percentage of responders in this group described the diet as problematic when going to a restaurant, family/friends’ house, or during traveling, compared with the “habitual” group [20]. Perhaps patients’ difficulties in following the low FODMAP diet stem from: Limited access to the recommended foods in regular supermarkets; the higher prices of products; insufficient information in the menu or lower palatability of new products. In our study, we did not examine detailed reasons for the perception of the diet as difficult to follow. Therefore, future studies should focus on identifying the challenges of and barriers to adhering to the FODMAP diet, which appeared to be more difficult for patients, and to explore why they defined it as less easy to follow compared to the NICE diet. 

One of the concerns raised about FAP is that the stress caused by the disease may significantly affect not only on children, but also parents [1,2,3]. Therefore, in our study, special attention was paid to the parental perception of the child’s symptoms during dietary intervention. The results showed that parents reported similar improvement in all symptoms of children in the low FODMAP group and NICE group. This outcome confirms previous findings in adult patients with IBS. Böhn et al. [22] and Eswaran et al. [23] reported a similar reduction of symptoms in both groups using the low FODMAP diet and traditional IBS dietary advice. 

The secondary endpoint focused on the examination of the agreement between parental and children’s assessments of gastrointestinal symptoms during dietary intervention. The result showed high agreement between parental and child perception of pain intensity and frequency. The percentage of compatible assessments for stool consistency was lower; the amount was less than half of the responses in both groups. It could be that a low agreement in stool consistency was due to the children’s embarrassment related to talking with parents about defecations, leading consequently to insufficient knowledge of parents. There are no studies examining the compatibility of the perception of symptoms between parents and children with FAPDs. Nevertheless, current literature studying chronic pain in children emphasizes the relationship between parent and child in pain perception. Firstly, parents, due to good knowledge of their child’s behavior, may easily assess the severity of pain [24,25]. Secondly, parental behavior and attitudes to the child’s disease may have a large impact on the level of pain perception by children. It was observed that parental overprotectiveness, worries or threat beliefs can increase pain catastrophizing in children [10,11,24]. Therefore, this issue should be investigated in future studies. Moreover, psychological consultation should be considered with both parents and children during dietary intervention to avoid the impact of this factor on the effectiveness of treatment.

This first study which evaluated the parental perception of the low FODMAP diet in children with FAP has several limitations. Firstly, due to the high availability of information about diets in the press or on the internet, parents may have suspected which diet their child was receiving. Hence, it cannot be concluded with absolute certainty that parents were blinded to the intervention. However, they were asked to limit conversations with children about information which might have suggested which diet they received. Secondly, the lack of a group without dietary intervention did not allow us to examine the potential placebo effect that could have occurred in each group. Another limitation is the fact that the parental assessment of children’s symptoms was recorded retrospectively on the summary meeting, and children reported their symptoms during the diet. A strength of our study was the use of a catering company to prepare and deliver meals to children. This allowed us to increase the control of the consumed products and standardized diets in groups. In addition, we evaluated parents’ perception of their children’s symptoms, and analyzed parental opinions about the diets. Those factors were not considered in any previous studies among children with FAPDs during dietary intervention. Lastly, clear dietary recommendations, provision of food product lists, and contact with the dietitian increased the effectiveness and satisfaction with the diet by parents and children.

## 5. Conclusions

In conclusion, this randomized, controlled, double-blind trial showed that in parental opinion the low FODMAP diet improved symptoms, as did the diet based on NICE guidelines in children with FAP. Moreover, these assessments had high compliance with symptoms reported by children. However, the low FODMAP diet was less often described by parents as an easy to follow diet, which may indicate the importance of monitoring and providing additional support by the whole therapeutic team during the intervention. Future research should focus on parents’ perceptions of therapy and clarity of recommendations because this may have impact on the effectiveness and acceptability of the diet by children.

## Figures and Tables

**Table 1:** Baseline characteristics of patients with functional abdominal pain.

	Low FODMAP Group	NICE Group	*p*
*N*	13	14	
Age *	7.9 ± 2.3	8.2 ± 1.9	0.721
Gender, female **	5 (39)	7 (50)	0.547
BMI z-score *	0.6 ± 1.0	0.3 ± 1.2	0.409
Children’s self-assessment of gastrointestinal symptoms **:			
Abdominal pain intensity			
Improvement	11 (85)	14 (100)	0.127
No improvement	2 (15)	0 (0)	
Abdominal pain frequency			
Improvement	8 (62)	13 (93)	0.050
No improvement	5 (38)	1 (7)	
Stool consistency			
Improvement	1 (8)	5 (36)	0.080
No improvement	12 (92)	9 (64)	

* Data are reported as mean ± SD; ** Data are reported as n (%).

**Table 2:** The assessment of the agreement of symptom perception between parents and children during dietary intervention.

Gastrointestinal Symptoms	Compatible Assessments		*p*
	FODMAP Group *n* = 13	NICE Group *n* = 14	
Abdominal pain intensity	11 (85%)	13 (93%)	0.496
Abdominal pain frequency	10 (77%)	12 (86%)	0.557
Stool consistency	3 (23%)	6 (43%)	0.363

Data are reported as n (%).

## Supplementary Materials

The following are available online at https://www.mdpi.com/1660-4601/17/15/5554/s1, Figure S1: The timeline of the study, Table S1: Sample daily meal plan of low FODMAP diet and diet based on NICE.
