# Supplementary material for: Parental Opinion about the Low FODMAP Diet in Dietary Treatment of Children with Functional Abdominal Pain
Source: Int J Environ Res Public Health. 2020 Jul 31;17(15):5554. doi: 10.3390/ijerph17155554 (PMC7432646; doi:10.3390/ijerph17155554)
Supplement: Supplementary file 1 [file ijerph-17-05554-s001.pdf]

International Journal of
Environmental Research and Public Health

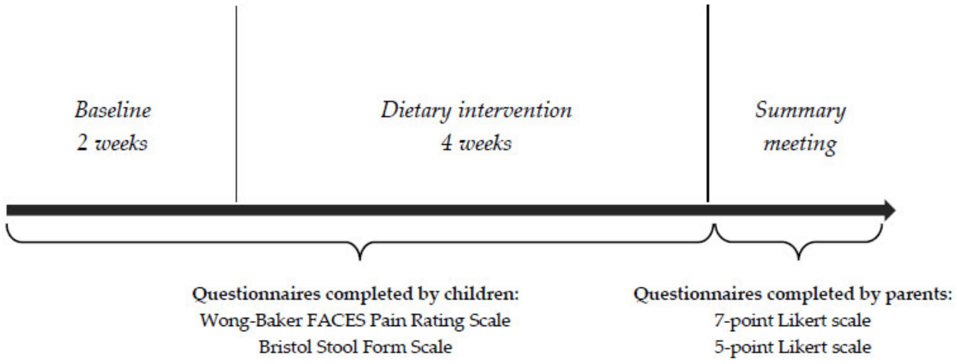

Figure S1. The timeline of the study.

Table S1. Sample of daily meal plan of low fermentable oligosaccharides, disaccharides, monosaccharides, and polyols (FODMAP) diet and diet based on National Institute for Health and Care Excellence (NICE).

Meal	Low FODMAP Diet		Diet Based on NICE Guidelines	
Breakfast	½ banana	green	½ banana	green
	Yoghurt, lactose-free	green	Yoghurt, natural	red
	2 tbsps. oatmeal, gluten-free	green	2 tbsps. oatmeal	yellow
	2 tbsps. puffed rice	green	2 tsps. pumpkin seeds	green
	3 tsps. sunflower seeds	green	1 tsps. sunflower seeds	green
	3 tsps. maple syrup	green	2 tsps. honey	red
	Cranberry juice with water and brown sugar	green	3 tsps. sultanas	red
			Tea with brown sugar	yellow
Lunch	30g turkey breast meat	green	30g turkey breast meat	green
	1/3 small zucchini	green	1/3 small zucchini	green
	½ small tomato	green	½ small tomato	green
	1 slice mozzarella cheese	green	1 slice mozzarella cheese	green
	1 tsp. olive oil	green	1 tsp. olive oil	green
	Water	green	Tea without sugar	yellow
Dinner	½ cup chicken soup	green	½ cup chicken soup	red
	1 bunch of sorrel	green	1 bunch of sorrel	green
	1 small potato	green	1 small potato	green
	½ egg	green	½ egg	green
	2 slices red pepper	green	2 slices red pepper	green
	2 slices orange pepper	green	2 slices orange pepper	green
	3 slices zucchini	green	3 slices zucchini	green
	1 tsp. mustard	green	1 tsp. mustard	green
	2 tsps. tomato paste	green	2 tsps. tomato paste	green
	2 tbsps. olive oil	green	2 tbsps. olive oil	green
	½ turkey sausage	green	½ turkey sausage	green
	2 small potatoes	green	2 small potatoes	green
	Cranberry juice with water and brown sugar	green	2 small mushrooms	red
			2 slices onion	red
			Cranberry juice with water and honey	yellow
Afternoon tea	30g orange	green	30g orange	green
	100g rockmelon	green	100g melon	red
	30g lemon	green	30g lemon	green
	100g pineapple	green	100g grapefruit	red
	2 tsps. maple syrup	green	2 tsps. maple syrup	green
Supper	¼ corn flour	green	¼ cup wheat flour	red
	1 tsp. olive oil	green	1 tsp. olive oil	green
	½ egg	green	½ egg	green
	¼ cup lactose-free milk	green	¼ cup low fat milk	red
	2 tsps. maple syrup	green	2 tsps. honey	red
	40g pineapple	green	40g mango	red
	40g papaya	green	40g pear	red
	Cranberry juice with water and maple syrup	green	Tea with brown sugar	green

FODMAP ratings: Red—high FODMAP; yellow—moderate FODMAP; green—low FODMAP; tbsp.—tablespoon, tsp.—teaspoon.
